# Autoimmune Pancreatitis Re-Classification with Novel Type AIP-4

**DOI:** 10.3390/ijms27093992

**Published:** 2026-04-29

**Authors:** Rolf Teschke

**Affiliations:** 1 Department of Internal Medicine II, Division of Gastroenterology and Hepatology, Klinikum Hanau, 63450 Hanau, Germany; rolf.teschke@gmx.de; 2 Academic Teaching Hospital of the Medical Faculty, Goethe University Frankfurt/Main, 60323 Frankfurt am Main, Germany

**Keywords:** autoimmune pancreatitis (AIP), AIP-1, AIP-2, AIP-3, AIP-4, AIP guidelines, sunlight exposure, secondary sclerosing cholangitis (SSC)

## Abstract

Autoimmune pancreatitis (AIP) represents a rare multifaceted disorder group with currently three types that were recently supplemented by a newly described AIP type causally related to sunlight exposure, not previously reported in any of the AIP publications. The internet search disclosed that the new AIP type was different from three existing AIP types: the AIP-1 or AIP-2 types, both featured by idiopathy, and the AIP-3 type, triggered by immune checkpoint inhibitors. Accordingly, it seemed appropriate to classify the novel AIP as the AIP-4 type, with typical features such as a clear culprit of sunlight exposure, ascertained by a positive result of an unintentional re-exposure and considered a diagnostic gold standard; coexisting secondary sclerosing cholangitis without progression to vanishing bile duct syndrome; and ultimately unavoidable pancreatic atrophy with clinical exocrine insufficiency despite long-term treatment with immunosuppressive drugs. Thus, the recent description of a new AIP type, now classified as the AIP-4 type, is strongly associated with significant sunlight exposure and calls for a reclassification of AIP types that includes AIP-4, whereby additional efforts are essential to identify the causative factors of the AIP-1 and AIP-2 types, including drugs commonly used in the respective cohorts, which also comprise older patients with comorbidities.

## 1. Introduction

Autoimmune pancreatitis (AIP) is a rare disease complex comprising several types termed AIP-1, AIP-2, and AIP-3 [1], now also supplemented by a recently described novel AIP type classified as AIP-4 [2], with characteristics substantially different from those of the previous three AIP types [1]. Although well investigated in the past, the external causative factors of the AIP-1 and AIP-2 types remained unknown [1,3,4,5,6]. In contrast, such idiopathy was not described for either the AIP-3 type [1] or the AIP-4 type [2]. Whereas the AIP-3 type is causally related to the treatment with the drug group of the immune checkpoint inhibitors (ICIs) in patients with cancer [7,8,9], the AIP-4 type is triggered by significant sunlight exposure, ascertained by unintentional re-exposure used as a diagnostic gold standard [2].

The heterogeneity of the previous three AIP types has resulted only in incoherent guidelines and fragmentary consensus recommendations regarding optimal diagnostic criteria and preferred therapies [5,10,11,12,13,14,15]. These shortcomings culminated in the view that an overall accepted consensus is not available, leaving many questions unanswered regarding diagnosis, causative factors, indications for treatment, and specifics of maintenance therapy, while emphasizing that only prospective randomized controlled trials (RCTs) are suitable to address the open questions [13]. These methodological concerns have not been abandoned in the past but are now even further augmented by the new AIP-4 [2].

The aim of this report is to provide an overview of current criteria for AIP-1, AIP-2, and AIP-3 that allow integration of the new AIP-4 type in future international approaches to guidelines and consensus recommendations. AIP-4 is a disruptive disorder with a focus not only on the exocrine pancreatic cell population but also affecting the cells of the biliary system and the hepatocellular cells. Conceptually and open to discussion, AIP-4 specifics include the role of sunlight in establishing the skin–pancreas axis, pancreas–biliary system axis, biliary system–liver axis, and skin–submandibular glands axis in support of active cross-talk among mediators and cell populations.

## 2. Clinical Features of the Novel AIP-4 Type Caused by Significant Sunlight Exposure

### 2.1. Positive Re-Exposure Test Confirms Cause

A prerequisite for a proposed new AIP type is a robust, evidence-based diagnosis with the new causative factor. The proposal of a novel AIP type classified as AIP-4 goes back to a report published early in 2026 describing two AIP flares of painless jaundice each following significant sunlight exposure [2]. The 76-year-old patient, a retired gastroenterologist, experienced in 2020 for the first time general jaundice in temporal association with gardening under significant sunlight exposure for 3.5 h, leading to visible sunburn, and was diagnosed with AIP based on a sausage-like appearance of the pancreas at imaging by computer tomography (CT), initially classified as AIP-2 due to normal serum IgG 4 values. The internet search revealed no AIP case in connection with sunlight exposure, leading to the clinical impression that the sunlight exposure of the patient was not related to his AIP but rather a factor of chance. In late 2023, however, general jaundice recurred following sitting outdoors in a restaurant under significant sunshine exposure for 2 h, leading to sun burn, interpreted clinically as second flare of the AIP, now ascertained by the unintentional re-exposure as a diagnostic gold standard and viewed as a strong argument for a causal association between the AIP and sunlight exposure. In view of these developments, the patient avoided, in the further course, direct sunlight exposures, resulting in normalization of all liver test results.

### 2.2. Laboratory Results Before and Under Therapy

The first flare of the AIP-4 was documented by abnormal liver test (LTs) of serum alanine aminotransferase (ALT), aspartate aminotransferase (AST), alkaline phosphatase (ALP), and gamma-glutamyl transferase (GGT) activities, associated with increased levels of serum total bilirubin (TBILI), but serum lipase remained surprisingly unaffected initially and later on [2]. To treat the first AIP-4 flare, the patient received prednisolone (PRED), prescribed as tablets of 40 mg daily for 4 weeks with subsequent tapering, in line with recommendations of the Japanese guideline of 2017 [10] and 2020 [11]. This regimen helped improve the initially increased LTs, but normalization was not achieved [2]. Therefore and in line with the Japanese guidelines [10] and Italian recommendations of 2017 [13], the patient received azathioprine (AZA) in daily doses of 100 mg, initially overlapping with PRED for 3.5 months, and later alone, until complete recovery of the LTs after an overall 2 years of treatment [2]. The LTs normalized intermittently but remained largely elevated during the long-term treatment period despite high AZA doses. The undulation of the LTs may have had several causes: (1) the AIP-4 patient also experienced a severe secondary sclerosing cholangitis (SSC) of the intrahepatic small bile ducts with stenoses and prestenotic dilatations not well accessible to the therapy used; (2) the patient might have benefitted if the treatment with PRED and AZA had been replaced by the immunomodulatory action of ursodeoxycholic acid to accelerate bile acid secretion, an option not discussed in earlier consensus recommendations nor in reports published in the years when the patient was under medical care; (3) the patient was under casual sunlight exposure because this risk factor was not recognized at the first AIP flare; and (4) notably, shortly after publication of the case report in early 2026 [2], the patient experienced another AIP flare but this time not related to sunlight exposure, successfully treated short-term with both PRED (40 mg/d) and subsequent URSO (3 × 250 mg/d), whereby the concomitant CT analysis revealed new calcifications of the aortic valve and the pancreatic head, This new flare was viewed as self-perpetuation of the AIP.

The liver injury pattern type was determined by calculating the ratio (R) value, as recommended by the updated Roussel Uclaf Causality Assessment Method (RUCAM) [16]. Accordingly, R was obtained using the multiples of the upper limit of normal (ULN) for ALT: ALP, whereby R ≥ 5 corresponds to hepatocellular injury, R ≤ 2 to cholestatic liver injury, and 2 < R < 5 to the mixed liver injury pattern. At the first flare, R was 0.22 (cholestatic) and at the second flare, R was 3.0 (mixed cholestatic–hepatocellular). Detailed laboratory results are presented in connection with a few case specifics (Table 1) [2].

### 2.3. Imaging Data

At hospital presentation as an out-patient on 13 February 2020, the abdominal ultrasound (US) examination of the patient showed an enlarged head of the pancreas of unclear nature, associated with a widened common bile duct of up to 11 mm and imaging features of intrahepatic cholestasis in an otherwise unremarkable liver, without suspected metastases [2]. The spleen and kidneys showed no abnormalities. The small intestine and colon were without abnormalities and showed more specifically no wall enlargement suggestive of pathological cockades as seen in Crohn’s disease or ulcerative colitis. The US examination of 15 October 2020 showed minimal intrahepatic cholestasis of the left liver with a normal appearance of the remaining parts of the liver; the common bile duct was unremarkable without dilatation or stenosis, while the head of the pancreas was marginally enlarged with pancreatic atrophy of the body and tail.

**Figure 1:**
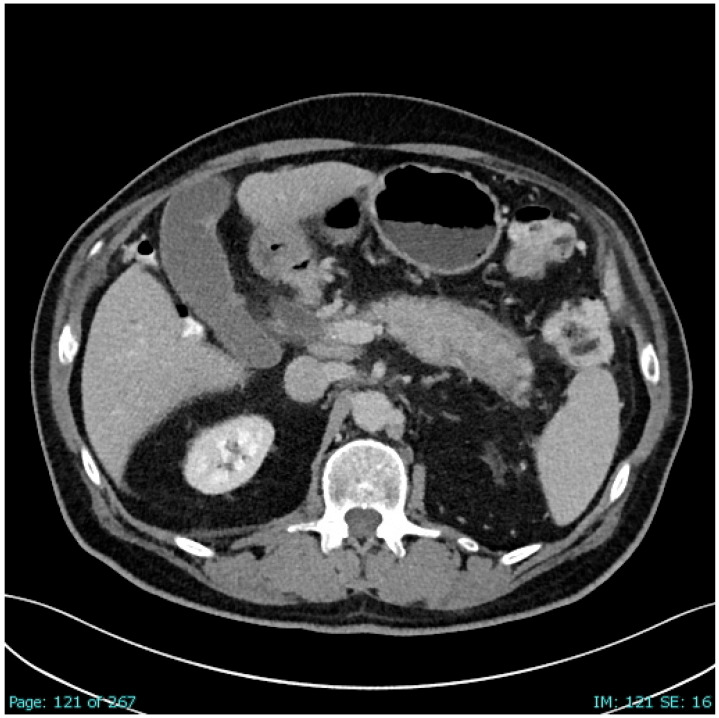
First CT scan of the patient with AIP-4. The computed tomography (CT) scan of the AIP-4 patient was obtained from a previous report published in an open-access journal [2]. The scan shows the portal-venous phase of the enlarged pancreatic tail with sausage-like appearance and a transverse halo sign in the middle right-sided of the scan, representing a scan that is typical and specific for autoimmune pancreatitis.

Because cancer of the pancreas head was suspected by US evaluation, a contrast-enhanced CT scan with contrast medium was undertaken. Among the most prominent CT findings was a substantial enlargement of the common bile duct up to 12 mm with a stop just above the entrance to the enlarged pancreas head, which led to luminal compression of the distal common bile duct [6]. All intrahepatic bile ducts appeared dilatated in an otherwise normal homogenous liver, without focal lesions such as metastases. The spleen, kidneys, and gastrointestinal tract were without pathological findings, excluding intestinal alterations in the sense of intestinal inflammatory disorders. The CT scan showed an enlarged head of the pancreas and a soft tissue tumescence around the body corpus and tail of the pancreas with a diameter of up to 8 mm in the sense of a sausage-like appearance strongly suggestive of AIP (Figure 1) [2].

**Figure 2:**
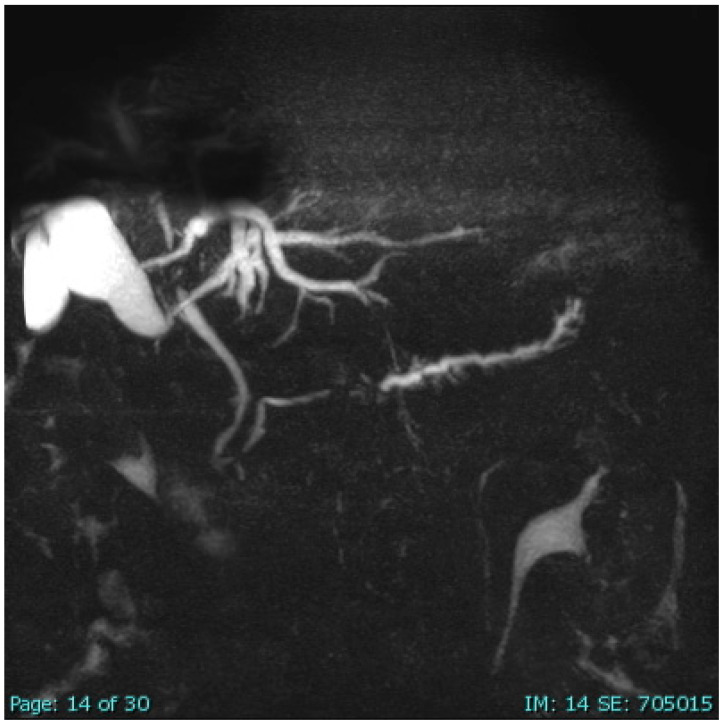
MRCP scan of the AIP-4 patient showing typical SSC signs of the small bile ducts. This MRCP scan of the AIP-4 patient was obtained from a previous report published in an open-access journal [2]. While the large bile duct and the lower common bile duct appeared normal, the upper common bile duct showed a circular configurated contrast uptake due to wall thickening, causing stenosing of the lumen. Similarly, the right and left intrahepatic bile ducts showed wall thickenings and stenoses in support of SSC associated with AIP-4.

The findings of the CT scan were confirmed by subsequent magnetic resonance cholangiopancreatography (MRCP), which again showed an edematous pancreas, compatible with AIP [2]. In the further course, 15 months after first presentation and while still under maintenance therapy with AZA, MRCP (native and with contrast medium) revealed, apart from a normal-appearing large bile duct and lower common bile duct, enlargement of the intrahepatic bile ducts, with substantial stenosis of the diameter above the hepatic fork. The upper common bile duct showed a circular contrast medium uptake due to wall thickening, causing stenosis of the lumen. Similarly, the right and left intrahepatic bile ducts showed a wall thickening and stenosis. These imaging chances observed 15 months after the first AIP-4 flare suggest newly developed secondary sclerosing cholangitis (SSC) in the context of the known AIP (Figure 2) [2].

The diagnostic work-up of the AIP-4 patient was expanded by the endoscopic ultrasonography (EUS) procedure describing an enlarged, poorly demarcated pancreas head of 28 × 22 mm, suspected to be carcinoma [2]. The concomitant EUS-fine needle aspiration of the pancreatic head revealed in the cytology aspirate several epithelial cells with eosinophilic cytoplasm but no cells suspicious for carcinoma. The esophago–gastroduodenoscopy provided negative results of helicobacter pylori by histology and the helicobacter urease test.

A recent review article summarized consensus and controversies surrounding AIP-2 [6]. The diagnostic consensus criteria for AIP-2 represent a quantitative diagnostic algorithm based on cardinal elements. Using the criteria that included imaging data of pancreatic parenchyma and duct, serology, other organ involvement, response to steroid therapy and pancreatic histology that cannot be replaced by cytology data [15], the diagnosis of the current case was provisionally determined probable. As the serum IgG4 level was normal in the patient (Table 1) [2], and because no cause was initially known, the case was provisionally aligned with the AIP-2 type defined by the criteria for both a normal serum IgG4 level and idiopathy [3,4,5,6]. However, sunlight exposure was finally recognized as a causative factor in the case (Table 1) [2], making the attribution to the AIP-2 type obsolete. Instead and considering all aspects, the patient was definitively diagnosed with the novel AIP-4 type [2] and viewed as different from the existing three AIP types [1].

### 2.4. Clinical Course

After all, xerostomia persisted, likely due to hyposalivation caused by exocrine salivary gland insufficiency, apart from disruption of the pancreas [2]. To overcome the consequences of exocrine pancreatic insufficiency, regular substitution with pancreatic enzymes was accomplished by capsules containing 25,000 units of lipase activity, 18,750 units of amylase activity, and 1,125 units of protease activity each [17,18]. In addition, vitamin D capsules (3500 µg each) were taken for 21 days to restore the lipid-soluble vitamin D levels. Prior to the first AIP flare, the body weight was 88 kg, which increased slightly to 90 kg under PRED treatment and decreased to around 81 kg after treatment with PRED was ceased. Otherwise, the clinical outcome after 5 years was fairly good following cessation of immunosuppressive drugs and attempts to avoid strict sunlight exposure, allowing only for little intermittent sunshine exposure to sustain vitamin D function.

### 2.5. Cross-Talk Open for Discussion

Circulating mediators as potential initiators of cross-talk among organs were determined in the AIP-4 patient only for serum IL-6 and were found to be increased [2]. This interleukin is known for its role in activating the transition between the innate and adaptive immune responses and helping recruit macrophages and lymphocytes to the site of the injury in autoimmune diseases [19]. Additional mediators were described in the serum of patients with AIP-1, focusing on INF-1, IL-33, IL-4, IL-5, IL-9, IL-10, and IL-13 [20] and in patients with AIP-2 with results of IL-17A, IL-21, IL-22, and IL-23 [3]. For both AIP types, the mediators are under discussion as pathogenetic factors for organ injury [3,18].

The AIP-4 is a multifaceted disorder because several organ systems are involved and functionally connected by organ–organ axes via assumed cross-talk of mediators secreted by cells of the organs of interest [2]. First of all, a functioning skin–pancreas axis will be operative, with mediators generated in the skin and transported as cellular disruptors via the systemic circulation to the pancreas as a target organ. Subsequently, a pancreas–biliary system axis must be operative and perhaps also a biliary–liver axis. The persisting xerostomia is likely due to a functioning skin–salivary gland axis. All steps are seemingly governed by the adaptive immune system linked to the innate immune system, which have to be activated by mediators. Similar axes are known for other immune disorders of internal medicine triggered by sunlight exposure; among these are porphyria cutanea tarda (PCT) with its skin–liver axis [21,22] and the special subtype 5 of Stevens–Johnson syndrome/toxic epidermal necrolysis (SJS/TEN) [23,24] in patients under drug therapy, resulting in skin lesions associated with liver injury attributed to cross-talk between the skin and the liver [25]. In the skin of these patients with SJS/TEN, lymphocytic inflammatory infiltration, high expression of granzyme, perforin, and FasL in mononuclear cells in TEN blisters were observed, suggesting CD8+ T-cell activation, which is likely responsible for epidermal keratinocyte necrosis [23,25]. At the molecular and immune level, sunlight exposure may lead to the generation of excess reactive oxygen species (ROS), whereby the cytotoxic effect of ROS in the skin triggers the immune system to attract T cells to the dermis. To expand this theory further, UV exposure increases vascular permeability, facilitating the passage of skin antigens into the bloodstream and favoring the formation of circulating antibodies and immune complexes [23,24,25].

## 3. Overview of Classification and Summarized Details of the Four AIP Types

In the past, many efforts focused on various types of AIP, which represent a heterogenous group of chronic pancreatitis due to organ disruption [1,2,3,4,5,6]. Current concerns and discussions focus on the harmonization of consensus recommendations and guidelines for diagnosis and therapy [2,4,5]. Some AIP types are rare conditions that impair evidence-based consensus recommendation of the best therapeutic options and make RCTs difficult. The AIP complexity and diversity have reached a higher level, as there are now four AIP types (AIP-1, AIP-2, AIP-3, and AIP-4), with different features that must be considered (Table 2) [1,2,3,4,5,6,7,8,10,13,20,26,27,28,29,30,31,32,33,34,35,36,37,38,39,40,41,42,43,44].

Serum autoantibodies in patients diagnosed with AIP-1 and AIP-2 remain a matter of debate due to their lack of specificity, which does not help define any of the currently known AIP types, as discussed in review articles from 2012 [45] and 2025 [46]. In more detail, a variety of autoantibodies have been identified in the sera of AIP-1 patients with IgG4 characteristics, as listed in a large compilation, considering autoantibodies that were either non-organ-specific or organ-specific, with a frequency in confirmed AIP-1 patients ranging from 12.5% to 100% [45]. However, the conclusion was reached that none of the autoantibodies appeared to be AIP-specific. With respect to the AIP-2 patients, anti-neutrophil cytoplasmic antibodies, anti-lactoferrin, and anti-carbonic anhydrase antibodies can be detected, especially in patients with concomitant ulcerative colitis, but the clinical relevance of these autoantibodies remains to be established [46]. There were no data on serum autoantibodies reported for AIP-3 [1,8,35,36,37,38,39,40,41,42,43,44,47,48,49] and AIP-4 [2].

## 4. Discussion

The overview of all four AIP types presents individual characteristics of each AIP type and provides sufficient evidence that the new AIP-4 type does not fit any of the three other AIP types (Table 2). The typical features of the AIP type are given in condensed form [2]: (1) significant exposure to sunlight verified as a new cause; (2) male gender, age 76 years, White ethnicity (European origin), born and living in Germany, genetic predisposition with respect to immune familial glaucoma; (3) lacking association with IgG4; (4) association with SSC; (5) the liver injury pattern was cholestatic at the first flare but it changed to a mixed hepatocellular–cholestatic type at the second flare; (6) mediators likely ensure cross-talk and help mediate the skin–pancreas axis, pancreas–biliary system axis, pancreas–liver axis, biliary system–liver axis, and skin–salivary gland axis; (7) PRED and AZA lack consistent therapeutic effects, and incorporating UDCA is likely a good therapeutic option; (8) disruption is responsible for the organ atrophy of the pancreas, leading to exocrine insufficiency, and of the salivary glands, causing persisting xerostomia based on exocrine insufficiency; (9) good news: there was no progression to vanishing bile duct syndrome; and finally (10), avoiding direct and significant sunlight exposure may prevent disease recurrence.

The features of the new AIP-4 type are clearly different from existing AIP types (Table 2) and will have to be adapted if additional AIP cases triggered by sunlight exposure are published. Conceptually and in the longer run, several AIP-4 subtypes may emerge, such as one confined to the pancreas, one combining pancreatic involvement with SSC, and one with a positive serum IgG4 pattern. Treatment options need refinement, considering perhaps early introduction of UDCA, especially in cases of persisting cholestasis or in associated SSC.

## 5. Conclusions

The introduction of the novel AIP-4 type calls for a future update of the current AIP classification, including AIP-4 characteristics. In the past, several classification updates were successfully achieved. Heterogeneity prevailed initially until AIP-1 was defined with serum IgG4 positivity and then distinguished from AIP-2, which is defined by IgG4 negativity. With the event of AIP caused by ICIs, the AIP-3 type was implemented in the AIP classification that now again requires refinement with inclusion of AIP-4. Based on the actual analysis of AIP reports and consensus recommendations, the impression prevails that a harmonized approach to these disruptive disease types is lacking, as shown by some selected issues requiring special consideration: (1) the diversity of national and international consensus recommendations for AIP-1 and AIP-2 cases is frustrating; (2) the heterogeneity of AIP cases related to geographical distribution, genetic predisposition, and gender ratio contributes to uncertainties; (3) missing straight forward efforts to clarify the causative factors of AIP-1 and AIP-2 is difficult to reconcile; (4) in particular, the unavailable information on drugs and herbal medicines in elder comorbid patients included in AIP-1 and AIP-2 cohorts neglects the role of medicines as possible causative factor factors; (5) of the lack of investigation into occupational exposure to chemicals as causative factors in AIP-1 and AIP-2 patients; (6) the absence of assessment of environmental factors such as recreational exposure to toxins or to sunlight in patients experiencing AIP-1 and AIP-2; (7) heterogeneity of AIP-1 cohorts due to inclusion of cases with serum IgG4 negativity that qualify as AIP-2; (8) similarly, heterogeneity of AIP-2 cohorts because cases with IgG4 negativity are included that clearly represent AIP-1 features; (9) inclusion of not otherwise specified (NOS) cases in study cohorts; (10) in patients with DILI induced by ICIs, expansion of the diagnosis may increase the number of AIP-4 cases with or without SSC; (11) AIP case heterogeneity with respect to the presence or absence of SSC as a critical factor for effective therapy; (12) RCTs for all AIP types should be carried out in major AIP centers; and, in essence (13) substantial efforts are needed to establish better definitions for each AIP category with its subtypes and to achieve harmonization of study cohorts that can help provide uniform therapeutic options.

## Figures and Tables

**Table 1:** Case details of the patient with two flares of the new AIP-4.

Dates	Case Details and Laboratory Data of the New AIP Type Termed AIP-4
13 February 2020 Prior significant sun exposure	**First flare in association with significant sunlight exposure** ALT 185 U/L; AST 97 U/L; ALP 302 U/L; GGT 447 U/L; TBILI 12.4 mg/dL; LIP 41 U/L; CA 19-9 149 U/mL; IgG4 0.333 g/L; IL-6 8.0 pg/mL. Normal serum ANCA and ANA titer. R 0.22.
16 February 2020	**Upon treatment with prednisolone (PRED) 40 mg/d, virtually all laboratory values improved** ALT 14 U/L; AST 24 U/L; ALP 66 U/L; GGT 70 U/L; TBILI 0.9 mg/dL.
18 February 2020	ALT 95 U/L; AST 42 U/L; ALP 250 U/L; GGT 320 U/L; TBILI 10.4 mg/dL.
4 March 2020	**Fluctuation of laboratory results necessitated switching from PRED to azathioprine (AZA), as evidenced by laboratory data** ALT 246 U/L; AST 108 U/L; ALP 168 U/L; GGT 142 U/L; TBILI 4.5 mg/dL.
1 April 2020	ALT 39 U/L; AST 27 U/L; ALP 90 U/L; GGT 62 U/L; TBILI 0.9 mg/dL.
6 May 2020	ALT 135 U/L; AST 72 U/L; ALP 257 U/L; GGT 411 U/L; TBILI 9.6 mg/mL.
10 June 2020	ALT 73 U/L; AST 32 U/L; ALP 116 U/L; GGT 256 U/L; TBILI 1.0 mg/dL.
15 July 2020	ALT 19 U/L; AST 24 U/L; ALP 72 U/L; GGT 62 U/L; TBILI 0.7 mg/dL.
23 September 2020	ALT 126 U/L; AST 59 U/L; ALP 176 U/L; GGT 1197 U/L; TBILI 1.4 mg/dL.
30 December 2020	ALT 12 U/L; AST 26 U/L; ALP 72 U/L; GGT 12 U/L; TBILI 0.7 mg/dL.
27 January 2021	ALT 14 U/L; AST 30 U/L; ALP 82 U/L; GGT 121 U/L; TBILI 0.9 mg/dL.
3 March 2021	ALT 16 U/L; AST 26 U/L; ALP 94 U/L; GGT 134 U/L; TBILI 0,7 mg/dL.
28 April 2021	ALT 14 U/L; AST 24 U/L; ALP 93 U/L; GGT 80 U/L; TBILI 1.0 mg/dL.
26 May 2021	ALT 28 U/L; AST 33 U/L; ALP 143 U/L; GGT 298 U/L; TBILI 1.0 mg/dL.
4 August 2021	ALT 15 U/L; AST 22 U/L; ALP 77 U/L; GGT 78 U/L; TBILI 0.9 mg/dL.
8 December 2021	ALT 19 U/L; AST 26 U/L; ALP 56 U/L; GGT 34 U/L; TBILI 0.5 mg/dL.
12 January 2022	ALT 23 U/L; AST 11 U/L; ALP 71 U/L; GGT 22 U/L; TBILI 0.5 mg/dL.
9 February 2022	ALT 11 U/L; AST 24 U/L; ALP 75 U/L; GGT 20 U/L; TBILI 0.6 mg/dL.
9 March 2022	**Treatment stop** ALT 12 U/L; AST 23 U/L; ALP 71 U/L; GGT 21 U/L; TBILI 0.5 mg/dL.
11 May 2022	ALT 15 U/L; AST 22 U/L; ALP 78 U/L; GGT 37 U/L; TBILI 0.5 mg/dL.
15 June 2022	ALT 13 U/L; AST 24 U/L; ALP 76 U/L; GGT 28 U/L; TBILI 0.7 mg/dL.
13 July 2022	ALT 8 U/L; AST 21 U/L; ALP 77 U/L; GGT 18 U/L; TBILI 0.7 mg/dL.
8 November 2022	ALT 15 U/L; AST 24 U/L; ALP 86 U/L; GGT 41 U/L; TBILI 0.5 mg/dL.
15 February 2023	ALT 17 U/L; AST 22 U/L; ALP 43 U/L; GGT 17 U/L; TBILI 0.6 mg/dL.
6 September 2023 Prior significant sun exposure +	**Last flare due to significant sunlight exposure** ALT 440 U/L; AST 310 U/L; ALP 470; GGT 77 U/L; TBILI 1.9 mg/dL; R 3.0. **Re-introduction of treatment with PRED combined with AZA**
11 September 2023 Sun exposure −	ALT 253 U/L; AST 119 U/L; ALP 314 U/L; GGT 777 U/L; TBILI 1.2 mg/dL.
27 September 2023 Sun exposure −	ALT 98 U/L; AST 92 U/L; ALP 275 U/L; GGT 505 U/L; TBILI 0.9 mg/dL.
24 January 2024 Sun exposure −	ALT 354 U/L; AST 217 U/L; ALP 479 U/L; GGT 638 U/L; TBILI 6.8 mg/dL.
21 February 2024 Sun exposure −	ALT 111 U/L; AST 58 U/L; ALP 199 U/L; GGT 319 U/L; TBILI 2.7 mg/dL.
10 April 2024 Sun exposure −	ALT 48 U/L; AST 34 U/L; ALP 95 U/L; GGT 73 U/L; TBILI 0.9 mg/dL.
12 June 2024 Sun exposure −	ALT 16 U/L; AST 24 U/L; ALP 53 U/L; GGT 24 U/L; TBILI 0.7 mg/dL.
21 August 2024 Sun exposure −	**Treatment stop** ALT 11 U/L; AST 19 U/L; ALP 57 U/L; GGT 17 U/L; TBILI 0.6 mg/dL.
26 March 2025 Sun exposure −	ALT 8 U/L; AST 18 U/L; ALP 74 U/L; GGT 17 U/L; TBILI 0.9 mg/dL.
21 May 2025 Sun exposure −	ALT 11 U/L; AST 20 U/L; ALP 79 U/L; GGT 17 U/L; TBILI 0.6 mg/dL.
15 October 2025 Sun exposure −	ALT 11 U/L; AST 21 U/L; ALP 77 U/L; GGT 16 U/L; TBILI 0.7 mg/d; HbA1c 5.9%; CA-19-9 8.1 U/L; CEA 2.7 U/L.

The table was taken from a previous open-access report [2]. The ratio (R) value based on the updated RUCAM [16] was 0.22 at the first flare, signifying a cholestatic liver injury pattern, and 3.0 at the second flare, signifying a mixed cholestatic–hepatocellular pattern. Normal ranges: ALT < 41 U/L; AST < 38 U/L; ALP < 129 U/L; HbA1c 4.5–5.7%; TBILI < 1.0 mg/dL; LIP < 60 U/L; CA 19-9 < 39.0, CEA < 3.4; IgG4 0.052–1.250; IL-6 < 7.0 pg/mL. The sign “−” means that sunlight exposure was thoroughly avoided if possible. Abbreviations: ALT, alanine aminotransferase; ALP, alkaline phosphatase; ANCA, antineutrophilic cytoplasmic antibody; AST, aspartate aminotransferase; AZA, azathioprine; CA 19-9, carbohydrate antigen; CEA, carcinoembryonic antigen; GGT, gamma-glutamyl transferase; IgG, immune globulin; IL, interleukin; LIP, lipase; PRED, prednisolone; TBILI, total bilirubin.

**Table 2:** The four AIP types: AIP-1, AIP-2, AIP-3, and AIP-4.

AIP Type	Triggering Factors, Genetics, SSC, Treatment, and Prevention	References
AIP-1	Triggering factors: unknown but yet poorly explored	Gallo, 2024 [4]
	Not yet explored as causative factors: drugs and herbal medicines	Okazaki, 2017 [10]
	Not yet explored: professional exposure to chemicals	De Pretis, 2017 [13]
	Not yet explored: occupational and recreational sunlight exposure	Nista, 2022 [3]
	IgG4 positivity in 90.64% but negativity in 9.36% like in AIP-2	Yamashida, 2025 [26]
	Geographical distribution: Asia > Europe and USA	Mack, 2022 [20]
	Mean age 65 years with male: female ratio of 3:1	Mack, 2022 [20]
	Systemic manifestations well described in summary form	Ohara, 2012 [27]
	Involvement of parotid and submandibular salivary glands	Nista, 2022 [3]
	Genetic predisposition: HLA-DRB1*0405-DQB1*0401, CTLA-4	Uchida, 2022 [28]
	Association with ulcerative colitis or Crohn’s disease (15–30%)	Nista, 2022 [3]
	Secondary sclerosing cholangitis (SSC) described	Saeki, 2025 [29]
	Treatment with ursodeoxycholic acid (UCDA) was effective	Masaki, 2021 [30]
	UCDA not mentioned in a recent comprehensive review on therapy	Tsubakio, 2002 [31]
	Relapse rate after PRED > 30%	Wu, 2025 [32]
	SSC at the first flare was a predictor of the second flare after 49 months	Saeki, 2025 [29]
	Pathogenetic role: INF-1, IL-33, IL-4, IL-5, IL-9, IL-10, and IL-13	Mack, 2022 [20]
	Mechanistic roles of the innate and adaptive immune systems	Nista, 2022 [3]
AIP-2	Triggering factors: unknown, but poorly explored	Gallo, 2024 [4]
	Not yet explored as causative factors: drugs and herbal medicines	Vemulapalla, 2025 [1]
	Not explored as firm causative factors: professional exposure to chemicals	De Pretis, 2024 [6]
	Not explored: occupational and recreational sunshine exposure	Zen, 2022 [5]
	IgG4 negativity in 90% but positive in 10% as in AIP-1	Zen, 2022 [5]
	Younger adults (30–40 years) without any gender preference	Ammer-Herrmenau, 2024 [33]
	Geographical distribution: Europe and USA > Asia	Mack, 2022 [20]
	Genetic predisposition under scientific discussion	Zen, 2022 [5]
	Localized rather than systemic manifestations	Zen, 2022 [5]
	Association with inflammatory disease in 40%	Zen, 2022 [5]
	SSC not described	Madhusudhan, 2019 [34]
	Treatment with PRED and AZA	Mack, 2022 [20]
	Relapse rate after PRED < 10%	Zen, 2022 [5]
	UDCA mentioned in a recent comprehensive review on therapy	Zen, 2022 [5]
	Pathogenetic roles of IL-17A, IL-21, IL-22, and IL-23 are discussed	Nista, 2022 [3]
AIP-3	Triggering factors: immune checkpoint inhibitors (ICIs)	Sayed Ahmed, 2022 [35]
	IgG4 levels may be normal in the serum but increased in the organ	Inoue, 2025 [36]
	IgG4 is commonly negative in serum but rarely positive like in AIP-1	Vemulapalli, 2025 [1]
	IgG4 levels in the serum are commonly in the normal range	Ito, 2024 [37]
	IgG4-rich plasma cells were not detected	Alsaleh, 3025 [38]
	Most patients were males (66%) with a mean age of 57 years	Abu-Sbeih, 2019 [39]
	Geographical distribution: mostly Japan (31/53) and Europe (16/53)	Pi, 2021 [7]
	Genetic predisposition: polymorphisms of CTLA-4 and PD-L1	Vemulapalli, 2025 [1]
	Systemic involvement as not typically described	Vemulapalli, 2025 [1]
	Bile duct injury in up to 19% of updated RUCAM-based DILI by ICIs	Hountondji, 2024 [8]
	UDCA alone effective in cholestatic RUCAM-based DILI by ICIs/SSC	Hountondji, 2025 [40]
	PRED treatment common, but its role and benefits remain unclear	Sayed Ahmed, 2022 [35]
	Holding ICIs may result in progression of the underlying cancer	Sayed Ahmed, 2022 [35]
	SSC in RUCAM-based DILI by ICIs was nonresponsive to PRED	Fontana, 2024 [41]
	UDCA treatment of choice in cases with SSC	Hountondji, 2025 [40]
	Pancreatic volume loss only with pancreatic enzyme elevation	Thomas, 2023 [42]
	Rapid organ volume loss despite treatment by PRED	Thomas, 2023 [43]
	Pathogenesis: CD8-positive T cells in bile ducts under discussion	Ogawa, 2019 [44]
AIP-4	Triggering factor: significant sunlight exposure prior to the two flares	Teschke, 2026 [2]
	IgG4 negativity with 0.333 g/L in the normal range	
	Male of 76 years and white ethnicity of European origin (Germany)	
	Genetic predisposition with respect to immune familiar glaucoma	
	Lack of systemic involvement of other organs far away	
	Initial RUCAM-based cholestatic liver injury pattern at first flare	
	At second flare, pattern change to the hepatocellular-cholestatic one	
	Cholestatic liver injury due to hepatocellular disruption by bile salts	
	Association with SSC characterized by typical MRCP results	
	Mediators ensure cross-talk among involved organs through axes	
	Skin–pancreas axis is the primarily functioning disruptive process	
	Pancreas–biliary system axis and pancreas-liver axis are important	
	Biliary system–liver axis is under discussion	
	Long-lasting treatment with PRED and AZA reduced elevated LTs	
	In cases with SSC, recommendation of switching to UDCA capsules	
	Continued pancreas disruption is not halted by PRED and AZA	
	Final exocrine pancreatic insufficiency due to organ atrophy	
	Xerostomia persisted likely due to impaired salivary gland function	
	Good news, finally normalization of cholestatic parameters	
	Good news, lacking transition to a vanishing bile duct syndrome	
	Finally, HbA1c was 5.9% (normal range 4.5–5.7)	
	Avoiding sunlight exposure as prevention is strongly recommended	

RUCAM-based data of causality and liver injury pattern were assessed in line with recommendations published earlier in the updated RUCAM report [16]. Abbreviations: AIP, autoimmune pancreatitis; AZA, azathioprine; DILI, drug-induced liver injury; PRED, prednisolone; R value, ratio; RUCAM, Roussel Uclaf Causality Assessment Method; SSC, secondary sclerosing cholangitis; UDCA, ursodeoxycholic acid.

## Data Availability

No new data were created or analyzed in this study. Data sharing is not applicable to this article.
